# Medically important *Candida* spp. identification: an era beyond traditional methods

**DOI:** 10.55730/1300-0144.5380

**Published:** 2022-03-02

**Authors:** Ebru EVREN, Jülide Sedef GÖÇMEN, Elvan HORTAÇ İŞTAR, Şükran YAVUZDEMİR, Alper TEKELİ, Yasemin YAVUZ, Zeynep Ceren KARAHAN

**Affiliations:** 1 Department of Medical Microbiology, Faculty of Medicine, Ankara University, Ankara, Turkey; 2 İbni Sina Education and Research Hospital Central Microbiology Laboratory, Faculty of Medicine, Ankara University, Ankara, Turkey; 3 Department of Medical Microbiology, Faculty of Medicine, TOBB University of Economics and Technology, Ankara, Turkey; 4 Department of Central Laboratory, Republic of Turkey Minister of Health Beytepe Murat Erdi Eker Public Hospital, Ankara, Turkey; 5 Department of Central Laboratory, Republic of Turkey Minister of Health Atatürk State Hospital, Zonguldak, Turkey; 6 Department of Biostatistics, Faculty of Medicine, Ankara University, Ankara, Turkey

**Keywords:** Candida identification, MALDI-TOF MS, CHROMagar *Candida*, corn meal tween-80 agar, sequencing

## Abstract

**Background/aim:**

*Candida* infections are gaining more attention for the last few decades so diagnostic tools are very important for early diagnosis. Conventional identification of yeasts is time-consuming, molecular methods are more complicated and relatively expensive gold-standard methods. Matrix-assisted laser desorption ionization time of flight mass spectrometry (MALDI-TOF MS) was put into the market due to its speed and high accuracy. The aim of this study was to evaluate the performance of corn meal tween-80 agar (CMTA), CHROMagar *Candida* medium, and MALDI-TOF MS and to compare the obtained results with DNA sequencing.

**Materials and methods:**

The CHROMagar *Candida* medium, CMTA, and MALDI-TOF MS Biotyper System were used to test 416 isolates. The isolates with discrepant results by at least one of the three methods were subjected to sequence analysis.

**Results:**

The identification results of the 351 (%84.4) were compatible with all three methods. When compared to the sequencing results, the most accurate results were obtained by the MALDI-TOF MS, especially for rare *Candida* species.

**Conclusion:**

MALDI-TOF MS is found to be the most accurate identification tool for clinically important *Candida* strains. CMTA alone should not be used for the final identification of *Candida* species and the chromogenic medium should always be considered presumptive.

## 1. Introduction

Fungal infections are gaining more attention for the last few decades, due to the increase in their incidences among susceptible hosts and their resistance characteristics [1]. Among them, *Candida* infections are the most prevalent ones. Although *Candida* strains are considered as nonpathogenic microbiological agents for immune-competent hosts, they are important causes of serious infections with high morbidity and mortality among immunosuppressed patients [2,3]. *Candida* species are capable of causing various clinical manifestations, among which bloodstream infections is one of the most important one associated with high mortality rates. Besides *C. albicans*, non-*albicans Candida* species also have a critical role in the pathogenesis. Morbidity and mortality of *Candida* bloodstream infections increase if the treatment is not appropriate and/or not immediate [4–6].

Due to the need for timely implementing of appropriate therapy, rapid diagnostic tools such as nucleic acid amplification methods and matrix-assisted laser desorption-ionization time of flight mass spectrometry (MALDI-TOF MS) are increasingly used for species identification. On the other hand, due to their high cost and unavailability for every diagnostic laboratory to install these techniques, conventional methods still remain the mainstays of the diagnostic approach [7–9].

Conventional identification of yeasts is time-consuming and depends on microscopy, germ tube test, and subcultures on corn meal tween-80 agar (CMTA; Dalmau culture) or on chromogenic media followed by biochemical tests. Reliable yeast identification can be achieved by assimilation reactions and fermentation tests, and commercially available kits can be used for this purpose. Additionally, MALDI-TOF MS was put into the market for the use of clinical microbiology laboratories about a decade ago, and since then it has been increasingly used due to its speed and high accuracy in the identification of main bacteria. Although its performance in fungal identification is somewhat lower than bacteria, it is still superior to commercial identification and is widely preferred for the identification of yeasts in clinical microbiology laboratories [10–12]. Molecular methods such as sequencing are considered the “gold-standard” for the identification of yeasts. On the other hand, these techniques are more complicated and relatively expensive [13]. The aim of this study was to evaluate the performance of CMTA, CHROMagar *Candida* medium, and MALDI-TOF MS for common and rare *Candida* species and to compare the obtained results with DNA sequencing.

## 2. Materials and methods

### 2.1. *Candida* isolates

A total of 416 *Candida* strains were evaluated in this study. The strains were isolated from cultures of different clinical samples such as a vaginal swab, tracheal aspirate, bronchoalveolar lavage fluid, catheter, and wound swabs and stored at −80 °C in tryptic soy broth with 20% glycerol. Although *Candida* spp. are part of normal flora and their presence in culture media should be regarded as an etiological agent with caution, all of the studied strains were considered as pathogens due to their clinical relevance as consulted with the clinicians and/or hospital infection control committee. Only one isolate from one patient was included in the study.

For the study, all of the stock cultures were subcultured on 5% sheep blood agar (SBA, Becton Dickinson, USA) for purity control. The following growth after 24–48 h incubation at 37 °C under normal atmospheric conditions, isolated colonies were subcultured on SBA for MALDI-TOF MS identification, CMTA (Oxoid, UK) for determining the *Candida* morphology and chlamydospore formation and CHROMagar *Candida* medium (Becton Dickinson, USA) for differentiation of *Candida* species according to the differences in colony color and morphology [11]. *C. albicans* ATCC 90028 and *C. parapsilosis* ATCC 22019 were used as the quality control strains.

### 2.2. CHROMagar *Candida* medium evaluation

The yeast colony was picked up with the tip of a sterile inoculating loop and plated on the surface of the CHROMagar *Candida* medium (BD, USA) by streak plate technique. The plates were incubated at 37 °C under normal atmospheric conditions and examined after 24 and 48 h by three independent investigators to identify *C. albicans* (light blue-green), *C. tropicalis* (metallic blue), *C. krusei* (pink, fuzzy). In addition, although it is not easy to discriminate, *C. glabrata* can often be distinguished by their specific colony colors as recommended by the manufacturer [11].

### 2.3. Corn meal tween-80 agar evaluation

A small amount of the yeast colony was picked up with the tip of a sterile inoculating loop and plated on the surface of the CMTA by making three consecutive parallel scratches. A flamed coverslip was placed over the inoculated scratches. The plates were incubated at 25 °C under normal atmospheric conditions and examined after 24, 48, and 72 h for the morphology and the production of chlamydospores by three independent investigators under the low power objective [8].

### 2.4. MALDI-TOF MS Biotyper system evaluation

Freshly grown yeast colonies were taken from the SBA plates with the tip of a wooden stick and smeared as a thin film onto MALDI target slides. The dried microbial film was then overlaid with 0.5-μL formic acid and 1.0 μL α-cyano-4-hydroxycinnamic acid (CHCA) MALDI-TOF MS matrix and air-dried for 1–2 min. at room temperature. The slide was inserted into the MALDI-TOF MS Biotyper system (Bruker Daltonics GmbH, Bremen, Germany) for data acquisition [11].

### 2.5. PCR and DNA sequencing analysis

The isolates with discrepant results by at least one of the three methods and randomly chosen forty isolates were subjected to sequence analysis. Total nucleic acid extraction was done by using the DNeasy Blood and Tissue kit (Qiagen, USA) according to the manufacturer’s instructions. The oligonucleotide primers ITS-1 (5′TTCGTAGGTGAACCTGCGG 3′) and ITS-4 (5′TCCTCCGCTTATTGATATGC 3′) were used to amplify an approximately 500 bp internal transcribed region sequence, as described previously [14]. Following PCR amplification, the products were purified and subjected to cycle sequencing by using both primers. Sequence analysis was performed by service procurement. The obtained electropherograms were visually controlled and the sequences were deposited to the GenBank for comparison and identification.

### 2.6. Statistical analysis

Compatibility of identification of each method was given by frequencies and percentages. Sequence analysis was considered the “gold standard” for the identification of yeasts and the compatibilities of the CHROMagar *Candida* medium, CMTA, and MALDI-TOF MS were calculated. McNemar χ2 test was used to compare compatibility between CHROMagar *Candida* medium, CMTA, and MALDI-TOF MS.

## 3. Results

Of the 416 strains, the identification results of the 351 (84.4%) were compatible with all three methods: The CHROMagar *Candida* medium, morphology, and chlamydospore formation on CMTA and MALDI-TOF MS system. *C. albicans* was the most widely identified yeast. The results of 65 (15.6%) isolates were discrepant by at least one of the three methods used in this study. The sequence analysis and the results of the overall agreements including compatible results indicating the methods used are given in Table.

Irrespective of the compatible results, besides *C. albicans, C. glabrata* is mostly identified strain. Other isolates such as *C. tropicalis*, *C. krusei, C. lusitaniae, C. parapsilosis*, and *C. inconspicua* are mostly identified species with three methods subsequent to *C. albicans* and *C. glabrata*.

When compared to the sequencing results, the agreements were differed according to the *Candida* species. The agreement of CHROMagar *Candida* medium, CMTA and MALDI-TOF MS Biotyper system for *C. albicans* was similar and greater than 90%, between three methods, CHROMagar *Candida* medium and MALDI-TOF MS Biotyper System have higher accuracy percentages than CMTA for the identification of *C. albicans*, however, the difference between percentages was not statistically different (p > 0.05). For *C. glabrata*, CMTA and MALDI-TOF MS system showed better agreement than CHROMagar *Candida* medium. For *C. kefyr, C. tropicalis, C. lusitaniae*, *C. parapsilosis*, and *C. inconspicua* only the MALDI-TOF MS system could identify the microorganisms at the species level.

Applying the McNemar X^2^ two-sided test, at the species level, there was no statistically significant differences were seen between CHROMagar *Candida* medium, CMTA and MALDI-TOF MS Biotyper System for *C. albicans, C. glabrata*, and *C. krusei* (p > 0.05). However, for *C. kefyr, C. tropicalis, C. lusitaniae, C. parapsilosis*, and *C. inconspicua* statistically significant differences were seen for MALDI-TOF MS Biotyper System (p < 0.001) which was superior to CHROMagar *Candida* medium (only for *C. tropicalis*) and CMTA.

## 4. Discussion

*Candida* spp. are the leading etiological agents of fungal infections. Until recently, *C. albicans* has been considered the most pathogenic yeast. However, non-*albicans Candida* strains are now increasingly reported as the causative agents of fungal infection making species-specific identification an important role of clinical microbiology laboratories both for epidemiological purposes and implementation of appropriate antifungal therapy. Widely known etiological agent *C. albicans* is the main opportunistic human pathogen, however *C. tropicalis, C. glabrata, C. krusei, C. parapsilosis* have also come into prominence [15]. Although infections caused by non-*albicans Candida* strains are highly similar to the *C. albicans* infections, virulence factors and antimicrobial susceptibility patterns differ from each other [8].

The interest in accurate identification had a historical value for more than 50 years. In a general aspect, there has been a shift from conventional methods to proteomics or nucleic acid amplification based methods in recent years, however, conventional diagnostic methods are still the mainstay of fungal infections because of their ease of use, low costs, and sometimes widespread use habits [16]. Reference and most widely used conventional procedures for *Candida* spp. identification include biochemical, morphological, and temperature studies. The disadvantage of these methods is the labor-intensity and the time-consuming pattern. Additionally, the conventional methods routinely used in the clinical microbiology laboratory for *Candida* spp. identification are not much accurate as compared to molecular methods and sometimes misdiagnose the species [17].

Identifying *Candida* species from culture includes germ tube test, chlamydospore formation, the fermentation/assimilation of sugars, and chromogenic media. In laboratory practice, the vast majority of the microbiology laboratories use at least one of the conventional methods mentioned above. However, none of these has 100% reliability; as a result, the most important issue is to discuss the misidentification possibility of the suspected colony by using any of the conventional methods. The germ tube is still used for rapid presumptive identification of *C. albicans*. Although rapid, the test is subjective, and less skilled laboratory personnel cannot differentiate a true germ tube from pseudohyphae. Moreover, the specificity of the test is reduced due to *C. tropicalis* that can produce early pseudohyphae that are mistaken for germ tubes and *C. dubliniensis*. Furthermore, 5% of *C. albicans* isolates do not produce germ tubes. Corn meal, CMTA, rice extract, and rice extract tween 80 agar are less subjective culture-based methods that are used for examining the chlamydospore formation microscopically. Besides, CMTA is also used in distinguishing the different species of *Candida* in slide cultures [16–18].

The microscopic examination on CMTA should be performed following the culture on another solid media such as SDA, making it a time-consuming method necessitating at least 72 h for final identification. It must be kept in mind that, species identification depending on morphology alone is not a reliable method for the identification of yeasts [18]. CMTA is usually recommended for the determination of chlamydospore formation and their relationship to hyphae, pseudohyphae and other fungal structures for probable identification and it only helps to narrow the genus range [7,18]. This medium was found to be successful in the identification of common *Candida* species except for the rare ones. Microscopic evaluation results are usually subjective and the decision on species identification depends on experience and species characteristics, and several factors such as incubation duration may influence morphological differentiation as well. Although Saeed et al. [19] tested CMTA for direct inoculation of the samples, they have found that CMTA can easily differentiate common *Candida* species; however, identification of rare ones is still challenging [20,21]. The results of our study are also similar to that of the previous study.

The need for identifying and discriminating *Candida* species as quickly as possible has led laboratories to use chromogenic media which allows *Candida* identification according to split the chromogenic substrates by species specific enzymes. Chromogenic media allows presumptive identification of some yeast species and it is used for identification of strains mostly encountered infectious diseases especially laboratories which do not use the MALDI-TOF MS system [22]. It can be used for the initial culture of clinical samples, making a quick presumptive identification possible. It is also good for the identification of mixed infections with “multi-species” yeasts as etiological agents. Chromogenic media are able to differentiate a limited number of *Candida* species such as *C. albicans, C. tropicalis*, and *C. krusei* [23,24]. According to previous studies, there is a lack of consensus for the identification of *C. glabrata* on CHROMagar *Candida* medium. Some authors claim that it can be differentiated from other species while some do not. The identification performance of *Candida* species with chromogenic media may depend on various factors such as the brands of the medium and even the sample obtaining site, as well [16]. Although *Candida albicans* remains as the principal agent of nosocomial yeast infections, many non-*albicans Candida* species, including *C. glabrata*, *C. parapsilosis*, *C. tropicalis*, *C. lusitaniae*, and *C. krusei*, have emerged as significant opportunistic pathogens and chromogenic media have the disadvantage of inability to differentiate certain important *Candida* species, even variability can be seen in terms of color and morphology for the same species [25]. Several reports claim that CHROMagar *Candida* medium can discriminate even *C. dubliniensis* from *C. albicans* and can be used for species identification [26–28].

Although Nerurkar V et al. [29] reported that CHROMagar *Candida* medium reliably identifies *C. albicans* and *C. tropicalis*, in our study the accurate identification rate for *C. albicans, C. glabrata*, and *C. krusei* was higher than *C. tropicalis*, and also only two of eight *C. tropicalis* were correctly identified by three methods. Vecchione A et al. [22] have reported that *C. albicans*, *C. tropicalis*, and *C. krusei* strains could be differentiated on the media following the manufacturer’s recommendations. In our study, the overall data showed that CHROMagar *Candida* medium correctly identified *C. albicans*, *C. glabrata*, and *C. krusei* 98.9%, 91.3%, and 64.3%, respectively. In clinical settings, rapid identification of *C. krusei* is especially important because of intrinsic resistance to fluconazole. In our study with the CHROMagar *Candida* medium, we have detected nine *C. krusei* isolates, however, only six were confirmed as *C. krusei* with the DNA sequencing method (the other three were *C. albicans, C. glabrata*, and *C. inconspicua*).

MALDI-TOF MS is an important technological evolution for the identification of microorganisms. It is widely used and adapted for bacterial identification, as it is easy to use, rapid, accurate, and cost-effective. The system is also a reliable method for the identification of *Candida* species-level identification [30]. Accurate species specific-level identification in clinical microbiology laboratories with mass spectrometry can vary according to the commercially available system used. For yeast species, various reports [31–33] demonstrate an accuracy rate between 90% to 96%, this also means that a portion with approximately 4%–10% can be omitted or misidentified. In our study, the data showed that the overall compatibility was also 98% at most even for *Candida albicans*. However, when we compared with other methods used in this study MALDI-TOF MS showed the best accuracy for yeast species identification included in this study. Accuracy rates of 100% were obtained for *C. tropicalis*, *C. parapsilosis*, and *C. inconspicua*, however, the low number of isolates was an important limiting factor. The important point for *C. kefyr* was that this species could only be 100% correctly identified with MALDI TOF MS. Although *C. kefyr* remains a rare cause of fungal infections and the epidemiology of this yeast is limited, Dufrense SF et al. [34] have reported that it is an emerging pathogen of serious concern, especially for patients with hematological malignancies. The diagnosis of this fungus will probably become more important in the following years.

A striking feature of this study is the inconsistent results for *C. albicans*, the most widespread cause of human pathogen. At least one method used in this study misdiagnosed 22 C. *albicans* isolates identified by sequence analysis. Chlamydospore formation is used to differentiate *C. albicans* from other *Candida* species with exception of *C. dubliniensis* and occasionally *C. tropicalis*. Chlamydospore formation can be influenced by many factors such as environmental conditions and genetic control. On the other hand, while chlamydospore formation can be observed mostly on solid media under glass coverslip providing oxygen- poor environment, chlamydosporulation is a complex process. *C. albicans* can be found in extreme variations and for this process, transcription factor Efg1 and various cellular pathways (MAP kinase, ...) have an influence on chlamydospore formation, and related gene defects can act as a negative factor for hyphae and chlamydosporulation and these processes also have an influence on protein expressions. Our data suggest that even *C. albicans* can be misidentified with common diagnostic tools which were enrolled in our study used in clinical microbiology laboratories [35–37]. As Pongrácz J et al. [38] reported resistance in clinical *Candida* isolates is a concern, accurate species identification is essential for the awareness of the intrinsic susceptibility profile of the isolate.

To conclude, MALDI-TOF MS is found to be the most accurate identification tool for clinically important *Candida* strains. Although sequencing is the gold standard for species identification, it is neither feasible nor economical for use in routine practice. Yeast characteristics and behaviors may be different and may be influenced by different factors. Phenotypic identification of yeasts depending on the colony and microscopic morphology is subject to these characteristics and to the expertise of the evaluator. This is the main limitation of using CMTA and CHROMagar *Candida* medium as the only identification tool for *Candida* species. CMTA alone should not be used for the final identification of *Candida* species. On the other hand, chromogenic media represent a valid possibility for rapid identification of the most frequently isolated *Candida* species directly from clinical samples and can be used for routine identification of frequently encountered yeast species in clinical laboratories that do not possess a MALDI-TOF MS and should always be considered as presumptive. For emerging rare *Candida* species, identification protocols and algorithms are gaining importance for clinical microbiologists.

## Figures and Tables

**Table:** The identification results of different methods and overall compatibility of CHROMagar *Candida* Medium, Corn meal tween-80 agar, and MALDI-TOF MS system results with sequence analysis.

	Compatible identification by three methods	Misidentified by at least one method but identified by sequence analysis	CHROMagar *Candida* medium	Corn meal tween-80 agar	MALDI-TOF MS Biotyper system	p-value
		n	n (%)	n (%)	n (%)	
*C. albicans*	**260/282**	22	279 (98.9)^a^	262 (92.9)^b^	277 (98.2)^c^	a,b,c > 0.05
*C. glabrata*	81/92	11	84 (91.3) ^a^	89 (96.7) ^b^	89 (96.7) ^c^	a,b,c > 0.05
*C. krusei*	8/14	6	9 (64.3) ^a^	9 (64.3) ^b^	12 (85.7) ^c^	a,b,c > 0.05
*C. kefyr*	0/15	15	NA*	0 ^b^	15 (100) ^c^	b,c < 0.001
*C. tropicalis*	2/8	6	0 ^a^	0 ^b^	6 (100) ^c^	a,b,c < 0.001
*C. lusitaniae*	0/3	3	NA*	0 ^b^	2 (66.6) ^c^	b,c < 0.001
*C. parapsilosis*	0/1	1	NA*	0 ^b^	1 (100) ^c^	b,c < 0.001
*C. inconspicua*	0/1	1	NA*	0 ^b^	1 (100) ^c^	b,c < 0.001
**Total**	**351/416**	**65**	**372**	**360**	**403**	

* NA: not applicable
